# Medication Reviews by a Clinical Pharmacist at an Irish University Teaching Hospital

**DOI:** 10.3390/pharmacy5040060

**Published:** 2017-10-27

**Authors:** Alan Kearney, Ciaran Halleran, Elaine Walsh, Derina Byrne, Jennifer Haugh, Laura J. Sahm

**Affiliations:** 1 Pharmacy Department, Mercy University Hospital, Cork T12 WE28, Ireland; challeran@muh.ie (C.H.); dbyrne@muh.ie (D.B.); jhaugh@muh.ie (J.H.); l.sahm@ucc.ie (L.J.S.); 2 Department of General Practice, School of Medicine, University College Cork, Cork T12 YN60, Ireland; elaine.walsh@ucc.ie; 3 The Pharmaceutical Care Research Group, School of Pharmacy, University College Cork, Cork T12 YN60, Ireland

**Keywords:** hospital pharmacy, pharmacist intervention, medication review, adverse drug event, Ireland

## Abstract

**Purpose:** Pharmacist-led medication reviews in hospitals have shown improvement in patient outcomes. The aim of this study is to describe the prevalence and nature of pharmacist interventions (PIs) following a medication review in an Irish teaching hospital. **Methods:** PIs were recorded over a six-month period in 2015. PIs were assessed by a panel of healthcare professionals (*n* = 5) to estimate the potential of adverse drug events (ADEs). Descriptive statistics were used for the variables and the chi square test for independence was used to analyse for any association between the variables. **Results:** Of the 1216 patients (55.8% female; median age 68 years (interquartile range 24 years)) who received a medication review, 313 interventions were identified in 213 patients. 412 medicines were associated with PIs, of which drugs for obstructive airway disease (*n* = 82), analgesics (*n* = 56), and antibacterial products for systemic use (*n* = 50) were the most prevalent. A statistically significant association was found between PI and patient’s age ≥65 years (*p* = 0.000), as well as female gender (*p* = 0.037). A total of 60.7% of the PIs had a medium or high likelihood of causing an ADE. **Conclusion:** Pharmacist-led medication review in a hospital setting prevented ADEs. Patients ≥65 years of age and female patients benefited the most from the interventions.

## 1. Introduction

Whilst medication is used to prevent, treat, and manage disease and illness, medication management is the most common intervention in order to prevent adverse drug events (ADEs) [1]. The use of medication has inherent risks, and medication errors compound these risks and can lead to increased morbidity and mortality [1]. The traditional role of a pharmacist as a compounder and a dispenser of medicines resulted in the pharmacist being quite detached from other healthcare professionals (HCPs) [2]. The profession has since evolved and the pharmacist is now recognised as an essential member of a multidisciplinary healthcare team [2]. The joint guidelines from the International Pharmaceutical Federation and the World Health Organisation (WHO) on good pharmacy practice have identified that multidisciplinary collaboration among HCPs is paramount to improving patient safety and outcomes [3]. In addition to sourcing, compounding, and dispensing medicines, pharmacists also provide tailored advice to both HCPs and patients on the optimal and safe use of medicines [2]. A pharmacist-led medication review and the communication of subsequent interventions to HCPs is an example of non-traditional pharmacy services provided by pharmacists [2].

Medication review is defined as “a structured, critical examination of a patient’s medicines with the objective of reaching an agreement with the patient about treatment, optimising the impact of medicines, minimising the number of medication related problems, and reducing waste” [4]. Medication review is a key element of medicines management to detect and reduce medical errors and to optimise medical treatment [4]. Due to their clinical knowledge and expertise, pharmacists are the ideal choice to undertake medication reviews [4,5]. A recent systematic review by Graabaek et al. identified that a pharmacist-led medication review in a hospital setting showed improvement in patient outcomes [6]. For our study, we adopted the following definition of a pharmacist intervention (PI): “any action taken by a pharmacist that aims to change patient management or therapy” [7,8].

Whilst medication reviews already occur within this hospital, there has been no official audit of what type of errors are occurring or their prevalence. This paper will describe the prevalence and nature of PIs following a pharmacist-led medication review in an Irish teaching hospital.

## 2. Methods

### 2.1. Setting

The study was undertaken in the Mercy University Hospital (MUH) which is a 350-bed general acute university teaching hospital in Cork in the south of Ireland. Data from this study cover a six-month time period from 1 May 2015 to 1 November 2015 inclusive. A hospital pharmacist reviewed patient’s medications prospectively, once or twice per week. Inclusion criteria were inpatients whose drug kardex was available for review. Patients were excluded if aged ≤17 years and those on specialty wards e.g., oncology, as they receive chemotherapy using a specific prescription form.

### 2.2. Intervention

The pharmacist-led medication review at the MUH consisted of a patient drug kardex review and, if required, was supported by the patient notes and laboratory data, but did not involve the patient as a source of data. When a PI(s) was identified, it was brought to the attention of the patient’s medical or surgical team for review.

### 2.3. Data Collection

Age, gender, type of care (medical/surgical), and length of hospital stay were collected for all patients. In addition, allergy status, co-morbidities, and the number of regular and “as required” (*pro re nata* (PRN)) medicines were recorded for patients with PI(s) only. A coding system was used to ensure confidentiality and data protection was guaranteed. The time taken for the pharmacist to conduct the medication review was also measured.

### 2.4. Classification of the PIs

PIs were classified by the hospital pharmacist according to type, based on the classification system employed by Gallagher et al. in a recent Irish study, but with minor modifications; the addition of two extra subheadings; duplication and poor prescribing practice, and the removal of rate of drug administration [8]. Poor prescribing practice reflects ambiguous prescribing which could be interpreted in more than one way, thus potentially affecting patient safety. Examples of these included illegible prescriptions.

The medicines associated with PIs identified were classified using the Anatomical Therapeutic Chemical (ATC) classification system [9].

### 2.5. Assessment of Potential Clinical Harm

PIs were reviewed and assigned a probability score, reflecting the likelihood of an ADE occurring in the absence of the PI, by five HCPs (three hospital pharmacists, an academic pharmacist and a general practitioner) using Table 1 as an example of how to assess potential clinical harm [8,10]. The median probability score for each intervention was used for analysis. An interrater reliability (IRR) analysis using the Kappa statistic was performed to determine absolute agreement between raters [11].

### 2.6. Data Analysis

Descriptive statistics were used to report the variables and the chi square test for independence was used to analyse for any association between the variables using the Statistical Package for the Social Sciences (SPSS) Version 20 (IBM Corp., New York, NY, USA).

The a priori level of statistical significance was set at *p* < 0.05. A test of normality of the continuous variables reported, patient age, length of hospital stay, number of co-morbidities, and number of regular and PRN medicines prescribed was performed. The chi-square test for independence was used to determine if there was a significant association between PI and patient’s age ≥65 years or patient gender. The Kappa measure of agreement was used to assess the strength of interrater agreement of assignment of probability scores to the PIs.

### 2.7. Ethical Approval

Ethical approval was granted by the Clinical Research Ethics Committee of the Cork Teaching Hospitals, University College Cork (UCC) and the local MUH management committee.

## 3. Results

### 3.1. Patient Characteristics

A total of 1216 patients received a medication review; PIs were identified in 213 patients. The demographics of the patients with and without PIs are displayed in Table 2.

A significant result for the Kolmogorov-Smirnov statistic (*p* < 0.05) for the continuous variables reported, patient age, length of hospital stay, number of co-morbidities, and number of regular and PRN medicines prescribed indicates that the data does not follow a normal distribution. A total of 843 co-morbidities were identified in patients with PIs with a median of 4 per patient and an interquartile range (IQR) range of 2. The most common co-morbidities identified included hypertension (*n* = 88), chronic obstructive pulmonary disease (COPD) (*n* = 74), dyslipidaemia (*n* = 56), cardiovascular disease (*n* = 54), and atrial fibrillation or flutter (*n* = 53). Those with PIs were prescribed a median of 11 regular medicines (IQR 7 medicines) per patient and a median of 2 PRN medicines (IQR 2 medicines) per patient.

### 3.2. PI Prevalence

A total of 313 PIs were identified in 213 patients. This represents an average of 0.26 PIs per patient who received a medication review(s), and an average of 1.47 PIs per patient who received a PI(s). The time taken for the pharmacist to conduct medication reviews was calculated as 180 h, which approximates to 0.19 Full Time Equivalent (FTE).

### 3.3. Types of PIs

Duplication, poor prescribing practice, frequency, dose, and interaction represents >70% of the PIs identified. The types of PIs and their prevalence are displayed in Table 3.

**Figure 1:**
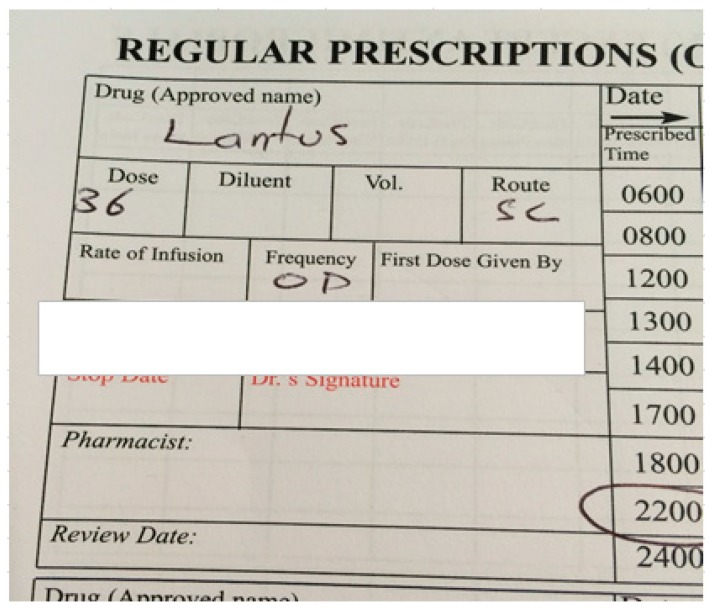
Example of unclear insulin dose.

Figure 1 displays a snapshot from a patient drug kardex reflecting poor prescribing practice.

### 3.4. Medicine Types Associated with PIs

A total of 412 medicine types were associated with PIs. The most common medicine types identified included drugs for obstructive airway disease (*n* = 82), analgesics (*n* = 56), antibacterial products for systemic use (*n* = 50), antithrombotic agents (*n* = 31), and drugs used in diabetes (*n* = 19).

### 3.5. Patient Characteristics Associated with PIs

A chi-square test for independence (with Yates continuity correction) indicated there was a significant association between: PI and patient’s age ≥65 years, χ^2^ (1, *n* = 1216) = 43.809, *p* = 0.000, phi = 0.192; and PI and female gender, χ^2^ (1, *n* = 1216) = 4.355, *p* = 0.037, phi = −0.062.

### 3.6. Potential for Adverse Drug Events

A total of 60.7% of PIs had a medium or high likelihood of causing an ADE. The prevalence and examples of PIs based upon the likelihood of an ADE occurring is displayed in Table 4. The Kappa measure of agreement values ranged from 0.114 to 0.324.

## 4. Discussion

This study describes the prevalence and nature of PIs following a pharmacist-led medication review in an Irish teaching hospital. A prevalence of 0.26 PIs per patient, who received a medication review, was found, which is within the range of 0.13 to 10.6, of the studies reported in a recent systematic review by Graabaek et al. [6]. The rate reported in this study was at the lower end of the scale which may be explained by time constraints, as the kardexes studied received a pharmacist-led medication review at a maximum of twice weekly. A total of 180 h was spent delivering the clinical pharmacy service during the six-month study period, which equates to approximately one fifth of a full time equivalent (FTE) of a hospital pharmacist. In comparison, in a similar study by Spinewine et al. that had 0.8 of a FTE of a clinical pharmacist, a higher PI rate was identified, indicating the time the pharmacist spends undertaking the intervention as a variable that influences the PI rate [13]. The number of PIs per patient who received a PI(s) was 1.47, which was lower than the 1.98 reported in a similar Irish study by Gallagher et al., who also reported that medicine omissions, relative to admission notes, represented 43% of the PIs compared to 4% of the PIs in this study [8]. Medication reconciliation, identifying medicine omissions relative to admission notes, was only opportunistically performed for patients in this study. This highlights that the definition used to categorise the intervention will influence the PI rate reported, indicating that a broader definition will lead to a higher PI rate [6].

Advancing age leads to an increase in the prevalence of chronic diseases and consequently drug consumption [14]. Increasing drug consumption together with patient metabolic changes with age predisposes older people to medication-related problems e.g., reduced elimination due to renal impairment, and ADEs [14,15]. Studies using the Screening Tools of Older People’s Prescriptions (STOPP) criteria have identified high levels of inappropriate prescribing in patients’ ≥65 years in the secondary care setting in Ireland [16,17]. Over three quarters (77%) of patients with PIs were ≥65 years of age in comparison to 52% of patients with no PIs. A statistically significant association was established between PI and patient’s age ≥65 years, which is in agreement with a recent systematic review by Graabaek et al., showing increasing age as an important variable [6].

Almost two thirds (62%) of patients with PIs were female, in comparison to 54% of patients with no PIs, leading to a statistically significant positive association. This is also supported by Krahenbuhl-Melcher et al., who identified female sex as a risk factor for ADEs or adverse drug reactions (ADRs) in hospitalised patients [18]. The reasons for the increased risk include gender differences in immunological and hormonal physiology that influence the pharmacodynamic and the pharmacokinetic response [19].

Duplication, poor prescribing practice, frequency, dose, and drug-drug interactions were the most common PI types identified. Medication for obstructive airway disease, analgesics, antibacterial products for systemic use, antithrombotic agents, and drugs used in diabetes were the most common medicine types associated with PIs. Despite the PI classification system for this study based on that employed by Gallagher et al., the prevalence of PI types and the medicine types associated with PIs differed between both studies [8]. These differences may be explained by the study population, design, and setting. In the study by Gallagher et al., medication reconciliation, identifying medicine omissions relative to admission notes, represented 43% of the PIs, and the study was undertaken across all wards of the hospital including the maternity unit [8].

Duplication, in this case the co-prescribing of a long-acting and a short-acting antimuscarinic bronchodilator as regular medicines, was the most common PI associated with drugs for obstructive airway disease in this study. The risk of a patient experiencing anticholinergic adverse effects is increased with combination therapy [20]. While the use of combination therapy may provide spirometry improvements in lung function, the clinical significance of these improvements has not been demonstrated [20]. Neither the British National Formulary 71st edition (BNF 71) nor the National Institute for Health and Care Excellence (NICE) clinical guideline for the management of COPD recommend concomitant use of long-acting and short-acting antimuscarinic bronchodilators [12,21].

Dose, in this case the prescribing of intravenous (IV) paracetamol at a dose of >60 mg/kg/day for patients <50 kg, and duplication, whereby paracetamol is prescribed as regular and PRN medicine with the cumulative dose exceeding the maximum daily approved normal dose, were the most common PIs associated with analgesics in this study. Paracetamol over dosage may result in hepatic injury, which could lead to hepatic failure requiring a liver transplant [22]. The risk of hepatic injury is increased in patients with hepatic impairment, in patients suffering from chronic alcoholism or chronic malnutrition, and in patients receiving enzyme-inducing drugs [12]. Annual reports available from the National Poisons Information Centre (NPIC), Ireland, identifies paracetamol as the most common drug involved in human poisoning (accidental and non-accidental) [23]. In Ireland, hospital pharmacists have responded to this danger by forming the Irish Medication Safety Network (IMSN) and have published a safety alert on the risks associated with IV paracetamol [24].

Drug-drug interactions, for example the inhibition of the cytochrome P450 metabolic enzymes by clarithromycin, was the most common PI associated with antibacterial products for systemic use in this study. Clarithromycin is a potent inhibitor of the cytochrome P450 3A4 metabolic enzyme [25]. In this study, the statins (3-hydroxy-3-methyl-glutaryl-coenzyme A reductase inhibitors) were the most common class that interacted with clarithromycin, with a subsequent risk of statin-induced myopathy [25]. This myopathy should be avoided where possible as it is an unpleasant and potentially debilitating experience and can lead to reduced patient compliance with possible discontinuation of therapy [26].

Anticoagulants are defined as a high alert medicine class in the acute care setting by the Institute of Safe Medication Practices (ISMP) in the United States of America (USA) [27]. Non-vitamin K oral anticoagulants (NOACs) are non-inferior and potentially superior to the vitamin K antagonist, warfarin, for stroke prevention in atrial fibrillation and for the prevention of venous thromboembolism [28]. NOACs are associated with lower rates of major bleeding, intracranial bleeding, clinically relevant but non-major bleeding, and total bleeding; however, with the exception of dabigatran, antidotes to reverse their effect are not available [29]. The prescribing of NOACs at subtherapeutic or supratherapeutic doses, as well as the timing and conversion between NOACs and parenteral anticoagulants and vice versa, were the most common PIs associated with antithrombotic agents in this study. NOACs require dose adjustment with changes in renal function and have a rapid onset of action therefore do not require overlap with parenteral anticoagulants [12,30]. Inappropriate use of the NOACs can lead to under anticoagulation or over anticoagulation, leading to increased risk of a thrombotic event or a haemorrhagic event, respectively. In Ireland, the IMSN has produced a safety alert on the risks associated with the NOACs [30].

Insulins are defined as a high alert medicine class in the acute care setting by the ISMP in the USA [27]. Insulin is a narrow therapeutic index medication and errors related to insulin are twice as likely to cause patient harm in comparison to errors involving other medications [31]. Poor prescribing practice, as evidenced by ambiguous dosing of insulin, was the most common PI associated with drugs used in diabetes in this study. The failure to specify any unit and the use of “U” as an abbreviation for “unit” were reasons identified. In the case of failure to specify any unit, the dose may be measured in terms of “unit”, “mL”, “mg”, or even “vial” or “pen”. The use of “U” as an abbreviation can lead to confusion as it can be mistaken for a trailing zero, leading to a ten-fold overdose. In Ireland, the IMSN has produced a best practice guideline on the safe use of insulin in Irish hospitals [32].

In the study by Gallagher et al., almost one fifth (19.73%) of the PIs were estimated to have a zero likelihood of causing an ADE, in comparison to a mere 0.32% of the PIs in this study [8]. Additionally, in the work by Gallagher et al., 28.7% of the PIs were estimated to have a medium or high likelihood of causing an ADE, in contrast to a much higher percentage of 60.7% in this study [8]. Differences in panel composition and the types of PIs identified may explain the results reported. As per Gallagher et al., the panel was composed of three academic pharmacists, and in this study the panel was composed of three hospital pharmacists, an academic pharmacist, and a general practitioner [8]. The high alert medicine class in the acute care setting published by the ISMP in the USA included two of the five most prevalent medicines types associated with PIs in this study, in comparison to none of the five most prevalent medicine types associated with PIs in the work of Gallagher et al. [8,27]. The results of our study have been presented at the Drugs and Therapeutics Committee and to the Chief Pharmacist of the MUH, with a view to gaining additional pharmacists who would undertake these medication reviews as part of usual care. In addition, a seminar for all prescribers on the key findings and implications for safe prescribing will be delivered biannually in January and July to coincide with the intern doctors’ educational programme. Medication reconciliation, identifying medication omissions relative to admission notes, was only opportunistically performed in this study. A formal medication reconciliation programme has proven to be successful for the patients of the geriatric ward and is planned to be rolled out to all wards in the hospital, once adequate resources are in place.

This study is not without its limitations, which include a single site, a small sample size, and the absence of a sample size calculation. The lack of a matched control group, to allow comparison, is a further limitation.

## 5. Conclusions

This study identified a prevalence of 0.26 PIs per patient who received a pharmacist-led medication review in an acute secondary care setting in Ireland. The results of this study confirmed previous work and supplemented the body of evidence that pharmacist-led medication reviews in a hospital setting reduce ADEs. Future studies may benefit from focusing on patients aged ≥65 years of age and female patients due the results observed in this study.

## Figures and Tables

**Table 1:** Probability scores with examples for the assessment of potential clinical harm of the PIs provided to raters.

Probability of ADE Occurring	Probability Score	Example
No harm expected	0	Pharmacist suggests changing a person from esomeprazole to omeprazole exclusively for economic reasons
Very low	0.01	Patient regularly takes a bisphosphonate, but medication omitted from hospital kardex
Low	0.1	Patient takes an antibiotic twice daily, when recommended dose would be three times daily
Medium	0.4	Metformin dose not reduced despite patient demonstrating renal impairment
High	0.6	Patient prescribed amiodarone while taking digoxin without any reduction in digoxin dose

ADE: adverse drug event.

**Table 2:** Demographics of study patients.

Demographic	Description	Patients with PI(s)	Patients with no PI(s)
		*n* = 213	*n* = 1003
Gender (n)	Male	80 (37.6%)	458 (45.7%)
	Female	133 (62.4%)	545 (54.3%)
Specialty (n)	Medicine	191 (89.7%)	820 (78.2%)
	Surgery	22 (10.3%)	229 (21.8%)
Age (years)	Median	74	65
	IQR	15	25
	≥65 years	*n* = 164 (77.0%)	*n* = 521 (51.9%)
Length of hospital stay (days)	Median	10.4	4.7
	IQR	11.4	6.9

PI(s): pharmacist intervention(s); *n*: number of patients; IQR: interquartile range.

**Table 3:** Types and prevalence of PIs (*n* = 313) in 213 patients.

Type of PI	No. of PIs (%)
**Duplication**	**87 (27.8%)**
Co-prescribe same drug class	45 (14.38%)
Co-prescribe same drug	42 (13.42%)
**Poor Prescribing Practice**	**41 (13.1%)**
Frequency of administration unclear	24 (7.67%)
Dose charted unclear	13 (4.15%)
Drug charted unclear	4 (1.28%)
**Frequency ^1^**	**38 (12.14%)**
More than approved normal frequency	21 (6.71%)
Less than approved normal frequency	17 (5.43%)
**Dose ^1^**	**33 (10.54%)**
More than approved normal dose	25 (7.99%)
Less than approved normal dose	8 (2.56%)
**Interaction ^1^**	**32 (10.22%)**
Pharmacokinetic	30 (9.58%)
Pharmacodynamic	2 (0.64%)
**Timing ^1,2^**	**27 (8.63%)**
**Review Therapy**	**23 (7.35%)**
**Omission ^3^**	**12 (3.83%)**
**Route**	**4 (1.28%)**
**Duration**	**3 (0.96%)**
**Other ^4^**	**13 (4.15%)**
**Total**	**313 (100%)**

PI: pharmacist intervention; PIs: pharmacist interventions. ^1^ As per Summary of Product Characteristics; ^2^ As per British National Formulary 71 [12]; ^3^ Relative to admission notes, this intervention was opportunistic; ^4^ For example, this includes medicines incorrectly transcribed.

**Table 4:** Prevalence and examples of PIs identified in this study based upon the likelihood of an ADE occurring.

Likelihood of an ADE Occurring	Number (%) of PIs ^1^	Example
Zero (no harm expected)	1 (0.32%)	Omeprazole prescribed as a PRN medicine with no doctor‘s signature
Very low	11 (3.51%)	Thiamine prescribed 200 mg OD as a regular medicine and two administration times circled
Low	111 (35.46%)	Combivent^® 2^ and Tiotropium both prescribed as regular medicines
Medium	169 (53.99%)	Solpadol^® 3^ II QDS prescribed as a regular medicine and Paracetamol 1 g QDS prescribed as a PRN medicine
High	21 (6.71%)	Enoxaparin and Rivaroxaban both prescribed as regular medicines

ADE: adverse drug event; PRN: “as required”; OD: once daily; QDS: four times daily. ^1^ This is based on taking the median probability score of the five healthcare professionals who rated the PIs; ^2^ Combivent^®^ is a combination product licensed in Ireland that contains ipratropium and salbutamol; ^3^ Solpadol^®^ is a combination product licensed in Ireland that contains paracetamol and codeine.
